# Median and small parsimony problems on RNA trees

**DOI:** 10.1093/bioinformatics/btae229

**Published:** 2024-06-28

**Authors:** Bertrand Marchand, Yoann Anselmetti, Manuel Lafond, Aïda Ouangraoua

**Affiliations:** Department of Computer Science, University of Sherbrooke, Sherbrooke, QC J1K 2R1, Canada; Department of Computer Science, University of Sherbrooke, Sherbrooke, QC J1K 2R1, Canada; Department of Computer Science, University of Sherbrooke, Sherbrooke, QC J1K 2R1, Canada; Department of Computer Science, University of Sherbrooke, Sherbrooke, QC J1K 2R1, Canada

## Abstract

**Motivation:**

Noncoding RNAs (ncRNAs) express their functions by adopting molecular structures. Specifically, RNA secondary structures serve as a relatively stable intermediate step before tertiary structures, offering a reliable signature of molecular function. Consequently, within an RNA functional family, secondary structures are generally more evolutionarily conserved than sequences. Conversely, homologous RNA families grouped within an RNA clan share ancestors but typically exhibit structural differences. Inferring the evolution of RNA structures within RNA families and clans is crucial for gaining insights into functional adaptations over time and providing clues about the Ancient RNA World Hypothesis.

**Results:**

We introduce the median problem and the small parsimony problem for ncRNA families, where secondary structures are represented as leaf-labeled trees. We utilize the Robinson-Foulds (RF) tree distance, which corresponds to a specific edit distance between RNA trees, and a new metric called the Internal-Leafset (IL) distance. While the RF tree distance compares sets of leaves descending from internal nodes of two RNA trees, the IL distance compares the collection of leaf-children of internal nodes. The latter is better at capturing differences in structural elements of RNAs than the RF distance, which is more focused on base pairs. We also consider a more general tree edit distance that allows the mapping of base pairs that are not perfectly aligned. We study the theoretical complexity of the median problem and the small parsimony problem under the three distance metrics and various biologically relevant constraints, and we present polynomial-time maximum parsimony algorithms for solving some versions of the problems. Our algorithms are applied to ncRNA families from the RFAM database, illustrating their practical utility.

**Availability and implementation:**

https://github.com/bmarchand/rna\_small\_parsimony.

## 1 Introduction

The RNA World hypothesis proposes that RNA preceded DNA as the primary genetic material on Earth (Bartel and Unrau 1999, Higgs and Lehman 2015). It suggests that RNA served as a carrier of genetic information and as a catalyst for chemical reactions, playing a central role in the early development of life before the evolution of the current DNA-based genetic system. Over time, DNA emerged as a more complex and stable molecule, taking over as the primary genetic material, while proteins became the catalysts for biochemical reactions.

An RNA molecule is mathematically defined as a string over an alphabet of four letters {A,C,G,U} corresponding to nucleotide bases. An RNA structure is defined by an RNA sequence and a set of canonical base pairs that form hydrogen bonds, resulting in the folding of the sequence. The structure of RNA molecules is closely linked to their function (Mattick 2005). Inferring ancestral RNA sequences and structures aims to understand how these structures have adapted and evolved over time, leading to the diversity of RNA observed today (Holmes 2004, Gruber *et al.* 2008, Tremblay-Savard *et al.* 2016). It can provide information about the RNA World hypothesis and the roles played by RNA in early biological processes by revealing patterns of conservation and providing insights into functional adaptations of RNA structures. Understanding the evolution of RNA structures also aids in reconstructing the evolutionary history of organisms.

Extensive work has been done on the reconstruction of ancestral sequences, which, given a phylogeny with observed sequences at its leaves, aims to find the sequences at the internal nodes and the changes that happened on the branches of the phylogeny, leading to the diversity of observed sequences (Fitch 1971, Sankoff 1975, Altschul and Lipman 1989, Schultz *et al.* 1996, Fredslund *et al.* 2003, Blanchette *et al.* 2008). To infer ancestral RNA sequences and structures, only two approaches have been proposed to date: In 2009, in a pioneering work, Bradley and Holmes (2009) developed a maximum likelihood approach to compute the ancestral RNA sequences and structures under the TKF Structure Tree model introduced in Holmes (2004). The TKF Structure Tree model is a continuous-time model of RNA structure evolution that extends the Thorne, Kishino, and Felsenstein’s 1991 (TKF91) model of sequence evolution Thorne *et al.* (1991). It allows not only single nucleotide substitutions and indels but also base-pair substitutions and indels, and whole sub-structure indels. However, the computational burden involved in the inference algorithm makes it prohibitive in practice. Then, Tremblay-Savard *et al.* (2016) developed a maximum parsimony method to compute the ancestral RNA sequences under a structurally constrained nucleotide substitution model, given an alignment of input sequences. The model allows only single nucleotide substitutions, and the method uses secondary structures as constraints. It considers a single consensus secondary structure for each RNA family and does not account for the structural differences between members of a family.

**Figure 1.**
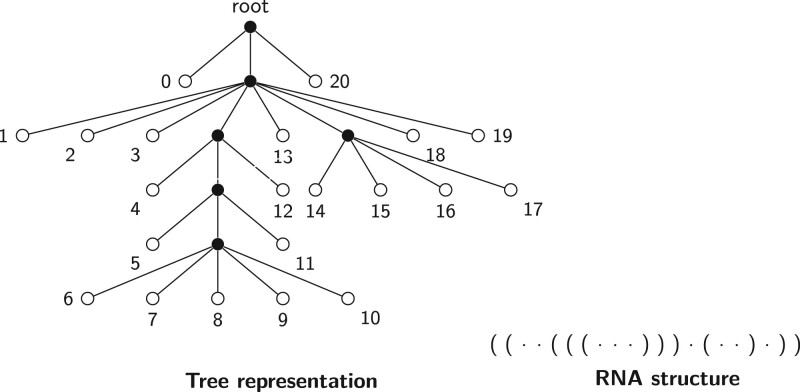
(Right) An RNA structure given in dot-bracket notation, and (Left) the corresponding tree representation (Definition 1).

In this article, we introduce the median and small parsimony problems on RNA trees. An RNA secondary structure is an RNA structure such that the RNA segments defined by any two pairs of bases forming hydrogen bonds are either strictly nested or disjoint. It is a hierarchical structure composed of paired and unpaired bases at the fine-grain level, defining structural elements such as loops and stems at the coarse-grain level. RNA secondary structures can be represented in the form of ordered rooted trees. There exist several different tree representations for RNA secondary structures depending on the level of representation, fine- or coarse-grain, and also depending on the representation of base pairs as single or multiple nodes in the tree representation (Le *et al.* 1989, Shapiro and Zhang 1990, Hochsmann *et al.* 2003, Ouangraoua *et al.* 2007). In this paper, we consider an RNA tree representation that extends the representation as a forest defined in Hochsmann *et al.* (2003). Leaf nodes correspond to bases in the 5ʹ to 3ʹ primary sequence order, and internal nodes correspond to bonds between paired bases. Each internal node has at least two leaf-children, the leftmost and rightmost children, which correspond to the paired bases linked by a chemical bond, plus potentially other leaf-children that form a loop in the secondary structure and other children that are internal nodes (see Fig. 1 for illustration).

**Figure 2.**
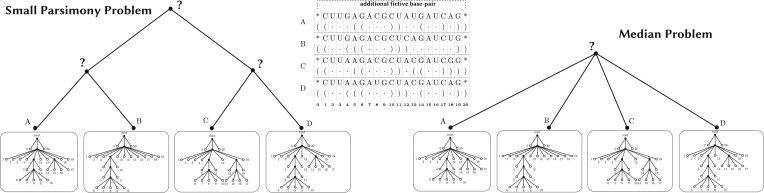
(Left) The small parsimony problem consists in finding the best assignment of RNA structures to internal nodes of a given phylogeny, minimizing the sum of distances over edges. (Right) The median problem consists in finding a single RNA structure that minimizes the sum of distances to the input structures.

We investigate two problems. Given a multiple RNA secondary structure alignment, the Median Problem involves finding an RNA secondary structure that minimizes the sum of distances to the input RNA structures. On the other hand, the Small Parsimony Problem, given a multiple RNA secondary structure alignment and a phylogeny on the input RNA structures, aims to determine an optimal assignment of RNA secondary structures at the internal nodes of the phylogeny, minimizing the sum of distances on the branches of the phylogeny. The Median and Small Parsimony problems on biological sequences are often considered together because they are related in the context of phylogenetics and ancestral sequence reconstruction. Many heuristic algorithms designed for the Small Parsimony problem involve starting with initial assignments of ancestral sequences at the internal nodes. The algorithms then iteratively improve the assignment at one node at a time by considering its neighboring nodes and solving median subproblems (Sankoff *et al.* 1976). The definition of both problems is illustrated in Fig. 2.

Here, we consider three distance metrics between RNA secondary structures, given a multiple RNA secondary structure alignment. We focus on the restricted case of gap-free alignments, equivalent to considering a set of RNA structures with the same sequence length. We thus assume that only base and base-pairs substitutions have occurred, and RNA sequences are not subject to indels, i.e. we consider the simplest algorithmic case where we do not handle base or base-pair insertions or deletions.

In the case where we have a gapped multiple RNA structure alignment, a gap-free alignment can be obtained by removing all gapped columns from the alignment. If only one side of a base pair in an RNA secondary structure is removed, the remaining side is marked as an unpaired base. The resulting RNA structures will then have the same sequence length.

The first metric we consider is the base pair distance, defined as the size of the symmetric difference between the sets of base pairs of two RNA structures. In a model of RNA structure evolution, the base pair distance can also be defined as the minimum number of base pair breaking or creation events required to transform one RNA structure into the other. We demonstrate that the base pair distance between two RNA trees equals the *Robinson-Foulds (RF) distance* between the corresponding RNA trees. The RF distance is a widely used metric for quantifying the dissimilarity between the topological structures of two phylogenetic trees (Robinson and Foulds 1981). Between two rooted trees on the same leaf set, it is defined as the size of the symmetric difference between the two collections of sets of leaves descending from internal nodes. The RF distance between phylogenetic trees does not have a direct biological interpretation in terms of specific genetic evolution, but between RNA trees, it does have a meaning in terms of RNA structure evolution in the case of gap-free multiple RNA structure alignments. It corresponds to a scenario of internal node deletion and insertion operations between two RNA trees, representing a scenario of base pair breaking and creation events between two RNA structures.

The RF distance essentially accounts for the difference in terms of the presence or absence of base pairings between two RNA structures. Yet, the difference in terms of loops is also relevant when comparing two RNA structures. Loops constitute the structural units at the basis of the stability of RNA secondary structures, as illustrated by the Turner Energy Model [Turner and Mathews (2010)], considered the gold standard of RNA secondary structure energy models. Within this model, the free energy of a structure is a sum of contributions over individual loops, depending on their precise nucleotide compositions. In this sense, the presence or absence of a loop directly contributes to the stability of the molecule. Inspired by the RF distance, we introduce a second distance metric called the *Internal-Leafset (IL) distance*, allowing us to account for the difference in terms of the presence or absence of loops. It is defined as the size of the symmetric difference between the two collections of leaf-children of internal nodes.

The base pair distance between RNA secondary structures is known to be very strict (Schirmer *et al.* 2014). We show that it can be expressed as the Tree edit distance between the corresponding RNA trees under a specific cost scheme. This cost allows the mapping between two base pair nodes of two RNA trees only if the base pairs are perfectly aligned in the multiple RNA structure alignment. By allowing the mapping between base pairs that are not perfectly aligned, we consider a third distance metric called the *Relaxed Edit (RE) distance*.

First, we present polynomial-time algorithms for computing the median of RNA trees under the RF, IL, and RE distances and various biologically relevant constraints on the output RNA tree. We then present polynomial-time exact and heuristic solutions for the small parsimony problem. Finally, we illustrate the application of our approaches on simulated RNA families and RNA families from the RFAM database (Lessa *et al.* 2012, Kalvari *et al.* 2021). For space reasons, proofs and figures are given in the Supplementary Appendix of the Supplementary material.

## 2 Preliminaries

### 2.1 RNA tree representation

An RNA secondary structure is represented by a pair R=(S,P) where *S* is a string of length *n* on the alphabet Σ={A,C,G,U}, and *P* is a set of pairs (*i*, *j*) such that 1≤i<j≤n and any $S_{i}S_{j}$ is either a Watson-Crick base pair (AU, UA, CG, GC) or a Wobble base pair (GU, UG). Any two pairs (*i*, *j*) and (*k*, *l*) in *P* satisfy one of the conditions: i<k<l<j, k<i<j<l, i<j<k<l, or k<l<i<j. We denote [i,j]={i,i+1,…,j}, which is empty if *i *>* j*.

We denote L(R)=[0,n+1] as the set of base positions [1,n] in *S* augmented with two fictive start and end positions, and *I*(*R*) as the set of pairs *P* augmented with a fictive pair (0,n+1), i.e. I(R)=P∪{(0,n+1)}. This ensures we have a tree representation of RNA structures (and not a forest). The union of these two sets is denoted by V(R)=L(R)∪I(R). The nesting of base pairs defines a partial order relation on *V*(*R*) denoted by ≺. For two pairs (*i*, *j*) and (*k*, *l*) in *I*(*R*), (i,j)≺(k,l) if and only if i<k<l<j. For a pair (i,j)∈I(R) and a base position k∈L(R), (i,j)≺k if and only if i≤k≤j.

The tree representing an RNA secondary structure R=(S,P) is an ordered rooted tree, denoted by *T*(*R*), with its set of nodes being *V*(*R*). The set of edges *E*(*R*) is determined by the nesting of base pairs. For two nodes *x* and *y* in *V*(*R*), there exists an edge (x,y)∈E(R) if and only if x≺y, and there is no third node z∈V(R) such that x≺z≺y. Thus, *I*(*R*) constitutes the internal nodes of the tree, with (0,n+1) being the root, and *L*(*R*) constitutes the leaf nodes. Moreover, for any internal node (i,j)∈I(R), the set of leaf nodes k∈L(R) descending from (*i*, *j*), i.e. such that (i,j)≺k, is [i,j]. The children of a node (i,j)∈I(R) are ordered according to the left-to-right ordering of the set of leaf nodes descending from them. Therefore, the leftmost and rightmost child of the node (*i*, *j*) are respectively the leaf nodes *i* and *j* (see Fig. 1).

Note that the partial order relation ≺ defined on *V*(*R*) is the ancestor-descendant relation on the nodes of *T*(*R*). For any two distinct nodes *x* and *y* in *V*(*R*), x≺y if and only if *x* lies on the unique path from the root to *y*. In this case, *y* is called a descendant of *x*. We further denote by < the total order on *V*(*R*) defined by a postorder traversal of the tree.

Definition 1 (RNA tree). An RNA tree is an ordered rooted tree *T* with an ordered leafset L=[k,l], 0≤k<l, such that for any internal node *x* of *T*, the leftmost and rightmost child of *x* are two distinct leaves *i*, *j*, in which case x=(i,j).

Given an RNA tree *T*, *L*(*T*) denotes its leafset, and *I*(*T*) its set of internal nodes. It is not difficult to see that RNA secondary structures and RNA trees are equivalent.

Proposition 1. *Given an RNA secondary structure*  R=(S,P)  *with S of length n, the tree T(R) is an RNA tree with leafset*  [0,n+1]*. Given an RNA tree T with leafset*  L(T)=[0,n+1]*, an RNA secondary structure*  R=(S,P)  *can be obtained by setting*  P=I(T)∖{(0,n+1)}  *and S as a string of length n on alphabet Σ such that for any pair*  $(i,j)\in P,\;S_{i}S_{j}$  *is either a Watson-Crick or Wobble base pair.*

Note that, given an RNA tree *T*, BP(T)=I(T) denotes the set of base pairs induced by *T*.

### 2.2 Distance between RNA trees

We consider two RNA secondary structures $R_{1}=(S_{1},P_{1})$ and $R_{2}=(S_{2},P_{2})$ of the same length *n* and their RNA tree representations $T_{1}=(V(R_{1}),E(R_{1}))$ and $T_{2}=(V(R_{2}),E(R_{2}))$. Note that the trees *T*_1_ and *T*_2_ have the same leafset $L(R_{1})=L(R_{2})=[0,n+1]$.

Definition 2 (Schirmer *et al.* 2014). The base pair (BP) distance between *R*_1_ and *R*_2_ is the size of the symmetric difference between *P*_1_ and *P*_2_: $d_{\text{BP}}(R_{1},R_{2})=|P_{1}\Delta P_{2}|=|I(R_{1})\Delta I(R_{2})|.$

Given a rooted tree *T* and an internal node *x* of *T*, the *descendant leafset* of *x*, denoted by DL(x), is the set of leaves *y* of *T* such that x≺y. In phylogenetics, DL(x) is also called a *clade*. The *internal leafset* of *x*, denoted by IL(x), is the set of children of *x* that are also leaves of *T*. We denote by DL(T), the collection of descendant leafsets of all internal nodes of *T*, DL(T)={DL(x)|x∈I(T)}, while IL(T) denotes the collection of internal leafsets of all internal nodes of *T*, IL(T)={IL(x)|x∈I(T)}. We consider the following distances between RNA trees *T*_1_ and *T*_2_.

Definition 3 (Robinson and Foulds 1981). The Robinson-Foulds (RF) distance between *T*_1_ and *T*_2_ is the size of the symmetric difference between $\text{DL}(T_{1})$ and $\text{DL}(T_{2})$: $d_{\text{RF}}(T_{1},T_{2})=|\text{DL}(T_{1})\Delta \text{DL}(T_{2})|.$

Definition 4 (Internal-Leafset distance). The Internal-Leafset (IL) distance between *T*_1_ and *T*_2_ is the size of the symmetric difference between $\text{IL}(T_{1})$ and $\text{IL}(T_{2})$: $d_{\text{IL}}(T_{1},T_{2})=|\text{IL}(T_{1})\Delta \text{IL}(T_{2})|.$

A *valid mapping M* between *T*_1_ and *T*_2_ is a bijection between a subset of $I(R_{1})$ and a subset of $I(R_{2})$ that preserves the order and the nesting relations of nodes. In other words, for any two pairs of mapped nodes (*x*_1_, *x*_2_) and (*y*_1_, *y*_2_) in *M*, we have $x_{1}<y_{1}$ if and only if $x_{2}<y_{2}$, and $x_{1}≺y_{1}$ if and only if $x_{2}≺y_{2}$. We denote by $M(T_{1},T_{2})$ the set of all valid mappings between *T*_1_ and *T*_2_. A cost function $c:I(R_{1})\times I(R_{2})\to R^{+}$ defines the cost of any couple in $I(R_{1})\times I(R_{2})$, such that $c(x_{1},x_{2})=0$ if $x_{1}=x_{2}=(i,j)$; otherwise, $c(x_{1},x_{2})>0$. Given a cost function *c*, the cost of a valid mapping *M* between *T*_1_ and *T*_2_ is then:

$$\text{cos}\;t_{c}(M)=\sum_{(x_{1},x_{2})\in M}c(x_{1},x_{2})+|I(R_{1})|+|I(R_{2})|-2\times |M|.$$

Definition 5 (Zhang and Shasha 1989). Given a cost function $c:I(R_{1})\times I(R_{2})\to R^{+}$, the Tree Edit (TE) distance between *T*_1_ and *T*_2_ under the cost function *c* is the minimum cost of a valid mapping between *T*_1_ and *T*_2_: $d_{{\text{TE}}_{c}}(T_{1},T_{2})=\min_{M\in M(T_{1},T_{2})}\;\text{cos}\;t_{c}(M).$

We can establish the following distance relationships.

Proposition 2 (Equality of BP distance and RF distance). *For any two RNA trees T_1_, T_2_*, $d_{\text{BP}}(R_{1},R_{2})=d_{\text{RF}}(T_{1},T_{2}).$

Proposition 3 (Equality of BP distance and TE distance under a specific cost function). *If the cost function c is defined as*  $c((i_{1},j_{1}),(i_{2},j_{2}))=0$  *if*  $\left(i_{1},j_{1})=(i_{2},j_{2}\right)$*, otherwise*  $c((i_{1},j_{1}),(i_{2},j_{2}))=+\infty$*, then*  $d_{\text{BP}}(R_{1},R_{2})=d_{{\text{TE}}_{c}}(T_{1},T_{2})$.

Definition 6 (Zhang and Shasha 1989). The Relaxed Edit (RE) distance $d_{{\text{TE}}_{c^{*}}}$ between *T*_1_ and *T*_2_ is the TE distance under the cost function $c^{*}:I(R_{1})\times I(R_{2})\to R^{+}$ defined as $c^{*}((i_{1},j_{1}),(i_{2},j_{2}))=|i_{1}-i_{2}|+|j_{1}-j_{2}|$.

### 2.3 Median and small parsimony problems

We now introduce the problems investigated in the article. We consider a set of RNA trees $T_{1},\ldots ,T_{p}$ with a common leafset L=[0,n+1]. Before introducing the definition of the problems, we first define some constraints that can be requested for the output RNA trees of the problems.

Definition 7 (Constraints on trees). An RNA tree *T* with leafset *L* is:

- Descendant-Leafset-Constrained (DLC) with respect to $T_{1},\ldots ,T_{p}$, if $\text{DL}(T)\subseteq \overset{p}{\underset{i=1}{\cup }}\text{DL}(T_{i})$
- Internal-Leafset-Constrained (ILC) with respect to $T_{1}\ldots T_{p}$, if $\text{IL}(T)\subseteq \overset{p}{\underset{i=1}{\cup }}\text{IL}(T_{i})$
- Not constrained (NC) if no constraint is applied.

Note that the DLC constraint allows to preserve another desirable constraint from the input trees $T_{1},\ldots ,T_{p}$ to the output trees, which is to have a lower bound *θ* on the number of bases that separate two bases *i* and *j* involved in a base pair.

**Problem 1** (D_C Median Problems).

**Input**: A set of RNA trees $T_{1},\ldots ,T_{p}$ with common leafset *L*; A distance $D\in \{d_{\text{RF}},d_{\text{IL}},d_{RE}\}$; A constraint on trees C∈{NC,DLC,ILC} with respect to $T_{1},\ldots ,T_{p}$.

**Output**: An RNA tree *T* with leafset *L*, satisfying the constraint *C* and minimizing

$$\mathrm{Mcost}(T)=\sum_{i=1}^{p}D(T_{i},T)$$

**Problem 2** (D_C Small Parsimony Problems).

**Input**: A set of RNA trees $T_{1},\ldots ,T_{p}$ with common leafset *L*; A distance $D\in \{d_{RF},d_{IL},d_{RE}\}$; A constraint on trees C∈{NC,DLC,ILC} with respect to $T_{1},\ldots ,T_{p}$; A tree T with leafset {1,…,p} such that each leaf *i* is assigned tree *T_i_*.

**Output**: A set of RNA trees $\left\{T_{x}|x\in I(T)\right\}$ assigned to internal nodes of T, each satisfying the constraint *C* and minimizing

$$\mathrm{SPcost}(\{T_{x}|x\in I(T)\})=\sum_{\left(u,v)\in E(T\right)}D(T_{u},T_{v})$$

To ease notation, in the problem statements, we may use RF, IL, RE instead of $d_{RF},d_{IL},d_{RE}$. For example, RF_DLC refers to the D_C problem with $D=d_{RF}$ and *C* = *DLC*. Note that in the D_C Small Parsimony problem, we do not require the input phylogeny T to be binary.

## 3 Computational complexity of the D_C medians

Given an RNA tree *T* with an ordered leafset L=[0,n+1], any descendant leafset of an internal node *x* of *T* is an interval of *L*, i.e. DL(x)=[i,j] with 0≤i<j≤n+1. Likewise, any internal leafset of an internal node *x* is a subset of *L*. We denote by DL(L) the set of all intervals of *L*, and by IL(L) the set of all subsets of *L*. We say that an RNA tree *T displays* a given DL *X* if there is a node *x* of *T* such that DL(x)=X. Similarly, *T displays* a given IL *I* if there is a node *x* of *T* such that IL(x)=I. Finally, *T* displays a given base pair (*i*, *j*) if (*i*, *j*) is an internal node of *T*.

### 3.1 RF_C medians

The main idea behind the algorithmic results for the Median Problems under the RF distance is that, given an ordered leafset L=[0,n+1], two DLs *X* and *Y* in DL(L) can be conflicting. Formally, *X* and *Y* are conflicting if they intersect, and their symmetric difference contains elements of both intervals. In this case, *X* and *Y* cannot be the descendant leafsets of two internal nodes of an RNA tree *T*.

The following property of conflict-free descendant leafsets is well-known for general rooted trees [see e.g. Theorem 3.1.4 in Semple and Steel (2003)]. It still holds for RNA trees.

Proposition 4 (Conflict-free descendant leafsets). *Given an RNA tree T*, DL(T)  *is conflict-free, i.e. any pair of descendant leafsets in*  DL(T)  *are not conflicting. Conversely, for any conflict-free subset*  DL′  *of*  DL(L)  *such that*  L⊆DL′*, one can find an RNA tree T such that*  DL′=DL(T).

Computing the median of a set of general rooted trees under the RF distance has already been solved in Barthélemy and McMorris (1986). The solution is also valid for RNA trees.

Proposition 5 (Majority-rule consensus tree). *Let*  ${\text{DL}}^{+}(T)$  *be the set of* DLs *displayed by strictly more than half of a set of RNA trees*  $T=\{T_{1},\ldots ,T_{p}\}$  *with common leafset L. Then there exists an RNA tree*  $T^{*}$  *such that*  $\text{DL}(T^{*})={\text{DL}}^{+}(T)$  *that is an optimal solution of the RF_NC Median Problem for*  $T_{1},\ldots ,T_{p}$*. Moreover, such a tree can be reconstructed in time*  $O(pn^{2})$.

Note that Proposition 5 also solves the RF_DLC Median, as all DLs displayed by $T^{*}$ are displayed by at least one tree *T_i_*.

### 3.2 IL_C medians

The majority-rule idea does not appear to be applicable to the IL Median Problems. Unlike DLs, there is no guarantee that one can construct an RNA tree that contains *exactly* the ILs that occur in more than half the trees. We thus develop a novel dynamic programming approach.

Note that an IL I∈IL(L) can always be expressed as a union of maximal intervals of *L*. We call *gaps* of *I* the set of intervals [i,j] of *L* such that none of [i,j] belongs to *I*, but *i* − 1 and *j *+* *1 belong to *I* (note that these are uniquely defined). We denote by Γ(I) the set of gaps of *I*. For integers *i*, *j*, we further define $I_{i,j}=\{I\in \text{IL}(L)|I\subseteq [i,j]\;\text{and}\;I\in \overset{p}{\underset{k=1}{\cup }}\text{IL}(T_{k})\}$, i.e. the set of ILs contained in [i,j] that occur in at least one input tree. Two distinct ILs *I* and *J* are *conflicting* if either I∩J≠∅ or ∃i,j⊆I and k,l⊆J such that i<k<j<l or k<i<l<j. If *I* and *J* are not conflicting, and min(I)<min(J)<max(J)<max(I), then all elements of *J* must be included in the same gap of *I*. We write $I{≼}_{IL}J$ when this is the case. Note that ${≼}_{IL}$ defines a partial order on IL(L).

We now define the concept of *structural partitions* of [i,j], with 0≤i<j≤n+1, which are the partitions of [i,j] that are in bijection with RNA trees with leafset [i,j].

Definition 8. A structural partition of [i,j] is a partition of [i,j] such that: (i) each set in the partition is of size at least 2 and (ii) the sets in the partition are not conflicting.

The following Lemma establishes a one-to-one correspondence between structural partitions and RNA trees.

Lemma 1. *Given an RNA tree T with leafset*  [i,j]*, the set*  IL(T)  *is a structural partition of*  [i,j]*. Conversely, given a structural partition*  I  *of*  [i,j]  *such that i and j are in the same set of the partition, there exists a single RNA tree T with leafset*  [i,j]  *such that*  IL(T)=I.

We next rewrite the cost function of a median, which will make it easier to design dynamic programming recurrences.

Lemma 2 (Rewriting cost function). *An RNA tree M is an IL median if and only if it minimizes*  $\sum_{I\in IL(M)}\left(\left|\{i|I\notin \text{IL}(T_{i})\}|-|\{i|I\in \text{IL}(T_{i})\}\right|\right)$.

For conciseness, for IL *I* we write $\text{cos}\;t_{IL}(I)=|\{i|I\notin IL(T_{i})\}|-|\{i|I\in \text{IL}(T_{i})\}|$. Note that the cost can be negative. The idea of the proof is that by the properties of the symmetric difference, the median must be penalized for its ILs not in the input trees (first term in the summation), but encourages the creation of ILs that are in those trees (second term in the summation). The main advantage of this rewriting is that it allows to break down the cost of a median as a sum of costs of individual ILs. That is, by Lemma 1, it suffices to find a structural partition of [0,n+1] of minimum sum-of-costs.

#### 3.2.1 Algorithm for the IL_ILC median problem

Recall that in IL_ILC, each IL of the median must be in the input. For i,j∈[0,n+1], we define

$$c[i,j]=\underset{{\;}_{\begin{array}{c} S\subseteq I_{i,j},\\ \;\text{structural partition of}\;[i,j] \end{array}}}{\min}\sum_{I\in S}cost_{IL}(I)$$

Note that we require $S\subseteq I_{i,j}$ because of the ILC constraint. Observe that c[0,n+1] represents the cost of an IL_ILC median (rewritten according to Lemma 2).

We define c[i,j]=∞ if $I_{i,j}$ is empty (in particular when i≤j). For general *i*, *j*, of course, there are too many structural partitions to enumerate, but we can use the fact that exactly one set *I* in the structural partition *S* must contain *i*. Since we constrain *I* to be in $I_{i,j}$, it suffices to enumerate in polynomial time every such possible IL, and to optimize each gap [x,y] independently. Note that if max(I)<j, then we must also optimize over the remaining gap [max(I)+1,j] (which could be empty). This leads to the recurrence:

$$c[i,j]={\min_{\begin{array}{c} I\in I_{i,j}\\ s.t\;i\in I \end{array}}}_{\;}\left[\text{cos}\;t_{IL}(I)+\sum_{\left[x,y]\in \Gamma (I)\cup \{[\max(I)+1,j]\right\}}c[x,y]\right]$$

By computing c[i,j] in increasing order of *j*−*i*, we eventually obtain c[0,n+1], which is the minimum cost of a structural partition, and backtracking can yield a median.

#### 3.2.2 Generalization for the IL_NC median problem

We next now show that the dynamic programming scheme may be generalized to the unconstrained IL_NC median. We define $\hat{c}[i,j]$ as the (unconstrained) structural partition of [i,j] of minimum cost, namely:

$$\hat{c}[i,j]=\underset{S,\text{structural partition of}\;[i,j]}{\min}\sum_{I\in S}cost_{IL}(I)$$

In order to enforce a minimum size of 2 for every IL, we set $\hat{c}[i,i]=+\infty ,\;\forall i\in [0,n+1]$. We also define $\hat{c}[i,j]=0$ if *j *<* i*.

The main difficulty in computing $\hat{c}[i,j]$ is that with the ILC constraint, there were few possible ILs in $I_{i,j}$ to enumerate for an entry c[i,j], but there are exponentially many possibilities after lifting the constraint. The main idea is to consider two cases: either the IL *I* that contains *i* is in $I_{i,j}$, or not. We already know how to handle the former case. In the case that *I* is not in any input tree, observe that every *I* that is not in $I_{i,j}$ has costIL(I)=p, the number of input trees. As we know that |I|≥2, we search for the smallest *k* that is part of the same IL as *i*. For such a *k*, we can use $\hat{c}[i+1,k-1]$ to optimize between *i* and *k* and optimize the gaps in [k+1,j]. There may be elements in [k+1,j] that are in the same IL as *i* and *k*, so we cannot simply invoke $\hat{c}[k+1,j]$ as this entry ignores the existence of *i* and *k*. Instead, we search for the optimal way to have gaps in the interval [k+1,j].

This generalization, therefore, relies on a polynomial-time subroutine solving the *maximum weighted independent set on interval graphs*. This subroutine will be called on a family of vertex-weighted graphs denoted $G_{k,l}$. More explicitly, $G_{k,l}$ has a vertex for every sub-interval [u,v], where k<u<v<l. Two sub-intervals [u,v] and [x,y] are connected by an edge in $G_{k,l}$ if they overlap, i.e. if [u,v]∩[x,y]≠∅. The weight associated with a vertex [u,v] in $G_{k,l}$ is $-\hat{c}[u,v]$, which is minus one times the minimum achievable cost over interval [u,v] (we multiply by –1 because the independent set is a maximization problem). An independent set of $G_{k,l}$ is a set of vertices that do not share any edge. The maximum weight of an independent set of $G_{k,l}$ is denoted $\alpha (G_{k,l})$.

As mentioned above, we minimize over the case where *I* is assumed to be in $I_{i,j}$ or not, which is computed in temporary entries ${\hat{c}}_{1}[i,j],{\hat{c}}_{2}[i,j]$, respectively, as follows:

$$\begin{array}{l} {\hat{c}}_{1}[i,j]=\underset{\begin{array}{c} I\in I_{i,j}\\ s.t\;\;i\in I \end{array}}{\min}\left[\text{cos}\;t_{IL}(I)+\sum_{\left[x,y]\in \Gamma (I)\cup [\max(I)+1,j\right]}\hat{c}[x,y]\right]\\ {\hat{c}}_{2}[i,j]=\underset{i<k\leq j}{\min}(p+\hat{c}[i+1,k-1]-\alpha (G_{k+1,j}))\\ \hat{c}[i,j]=\min({\hat{c}}_{1}[i,j],{\hat{c}}_{2}[i,j]) \end{array}$$

For the computation of ${\hat{c}}_{2}[i,j]$, *p* represents the cost of the IL that contains *i* and *k*, $\hat{c}[i+1,k-1]$ is the cost of the gap between *i* and *k*, and $-\alpha (G_{k+1,j})$ the cost of the gaps to the right of *k*. See the Supplementary Appendix for a detailed pseudo-code. Using the algorithm of Hsiao *et al.* (1992) for independent sets in interval graphs, we obtain the following.

Theorem 1. *An IL_ILC median can be found in time in*  $O(p\cdot n^{3})$*, where p is the number of input trees and n is the number of leaves in the input trees. Moreover, an IL_NC median can be found in time*  $O(p\cdot n^{5})$.

In fact, Theorem 1 is valid for the RF_ILC median problem under a slight change of the cost function that is, minimizing the RF distance while requiring every IL to be in the input.

##### 3.2.2.1 RE_C Medians

The approaches used above cannot be applied to the RE distance. Indeed, because different base pairs of the median can be mapped to different base pairs of the input trees, no majority rule seems applicable as in the RF case. For the same reason, the cost of a median cannot be expressed as a sum of independent costs for each base pair as in the IL case.

Therefore, the complexity of the RE_C Median Problems remains open.

## 4 Complexity of D_C small parsimony

### 4.1 RF_C small parsimony

We give here a polynomial-time algorithm for the unconstrained RF_NC Small Parsimony. The algorithm is largely inspired by the one from Feijao and Meidanis (2011), which solves the small parsimony for genome rearrangements under the SCJ distance. Interestingly, when genomes are specified as lists of *adjacencies*, they also have a notion of *conflict* that applies in the same manner as DL conflicts apply to trees. That is, a set of adjacencies corresponds to a genome if and only if they are conflict-free. This is analogous to the fact the a set of DLs corresponds to a tree if and only if they are conflict-free. We remark that the algorithm given in Feijao and Meidanis (2011) does not depend on the particular notion of conflict they use, and can therefore be adapted to our case. The idea is to view each possible DL as present (1) or absent 0 in each leaf, and to execute the Fitch-Hartigan algorithm on the 0−1 representation of the DLs (see Fitch 1971, Hartigan 1973). As it turns out, we can avoid conflicts by choosing a 0 at the root whenever the algorithm gives us a choice between 0 and 1. Details are presented in the Supplementary Appendix. In summary, they show that starting from conflict-free sets of DLs at the leaves, and applying the two passes of Fitch-Hartigan’s algorithm propagates this property and leads to an optimal solution.

Theorem 2. *The Small Parsimony for the RF_NC and RF_DLC distances can be solved in time*  O(pn|V(T)|)*, where p is the number of RNA trees and n their number of leaves.*

### 4.2 IL_C small parsimony

The above approach for the Small Parsimony under the RF distance cannot be applied immediately to the IL distance. Indeed, in the RF case we could use the algorithm of Feijao and Meidanis (2011) because, for any set of nonconflicting DLs inferred at internal nodes, we can construct an RNA tree with exactly that set of DLs (in fact, the algorithm works for any set of objects with this property). In the case of ILs, it is not true that for any set of nonconflicting ILs, we can build a tree with exactly those ILs. In particular, the set of ILs of a tree must form a partition of [0,n+1], which is not guaranteed by the previous algorithm.

Since the IL median can be computed in polynomial time, we can instead make use of the popular median-based heuristic for the Small Parsimony problem (Sankoff *et al.* 1976). That is, we can proceed as follows: (i) infer an initial set of ancestors in T [e.g. by requiring that each ancestor is assigned one of the RNA trees that appears at the leaves while minimizing the *SPcost*, which can be done in polynomial time using the classical dynamic programming of Sankoff and Rousseau (1975)]; (ii) for each nonroot internal node *u* of T, find the IL median of the RNA trees assigned at the neighbors of *u* (this includes the parent, so there are at least three neighbors); (iii) if assigning the median to *u* improves the total cost on the branches of T, then re-assign *u* to the median; (iv) repeat until no such improvement is possible.

### 4.3 RE_C small parsimony

The approach inspired by Feijao and Meidanis (2011) cannot be applied to the RE_C Small Parsimony either because the Fitch-Hartigan method cannot be applied to compute a minimum presence/absence (0/1) assignment for each base pair, independently of other base pairs. Indeed, two different base pairs from two distinct input trees can be mapped in an optimal assignment of RNA trees to ancestral nodes. Therefore, base pairs cannot be considered independently of each other. The complexity of the RE_C Small Parsimony remains open.

Although we know of no good algorithm for the RE_C Small Parsimony or Median problems, we can infer an assignment of RNA trees to ancestors in the phylogeny T, with the requirement that each ancestor is assigned one of the RNA trees that appears at the leaves, and the *SPcost* of the assignment is minimal as in the first step of the previous IL_C heuristic.

## 5 Experimental results

We implemented the algorithm underlying Theorem 2 (exact solution for the RF_NC Small Parsimony problem) as well as the median-based heuristic described above (Section 4.2) for the RF_ILC, IL_ILC and IL_NC versions of the Small Parsimony problem. As initial assignment, we compute the best internal node assignments when restricted to the trees present at leaves. We also used this assignment as the whole heuristic for the RE distance (Section 4.3). All of the code is available at https://github.com/bmarchand/rna\_small\_parsimony.

We could not compare the results of our method to the two existing approaches cited in the introduction, namely Bradley and Holmes (2009) and Tremblay-Savard *et al.* (2016). Unfortunately, the source code for Bradley and Holmes (2009) is no longer available at the time of writing this paper. As for Tremblay-Savard *et al.* (2016), the method computes ancestral sequences while our methods compute ancestral structures.

### 5.1 Datasets

These algorithms were tested on two sets of data. The first one is RFAM [version 14.10, Kalvari *et al.* (2021), https://rfam.org/], the leading database for structured RNAs. It regroups RNA sequences in families of structural homologs, for which it provides a manually curated multiple alignment (seed alignment) with consensus structure annotation, and a phylogeny. We used our algorithms to infer ancestral structures for all internal nodes of these single-family phylogenies. For each sequence of each family, the consensus structure of the RFAM alignment was first restricted to Watson-Crick and Wobble base-pairs. Then, it was augmented using RNAfold (Lorenz *et al.* 2011), under the constraint that all base-pairs from the consensus annotation had to be kept.

Note that not necessarily all base-pairs in the consensus are valid (i.e. pair up compatible nucleotides) for all sequences in the family. Only such valid base-pairs were kept as constraints when applying RNAfold. Then, a gapless alignment was obtained for each family by removing from the seed alignment any column containing a gap. When within the structural annotation of a sequence, a paired position is deleted this way, its partner is considered unpaired. After this pre-processing applied, only families whose sequence lengths were ≤100 were selected for our experiments, amounting to 2936 RNA families (out of 4170 in Rfam 14.10). Within them, 1639 families exhibit after this process the same structural annotation for every sequence, and are therefore excluded. Combined with the phylogenies provided by RFAM for each family (in which each sequence of the seed alignment corresponds to a leaf), the remaining 1297 families form what we will now refer to as the FILTERED_RFAM dataset.

We also tested our algorithms on complete binary phylogenies (of height *H *=* *5) annotated with random secondary structures at leaves. Structures were drawn from the uniform distribution over the set of admissible secondary structure annotations of length *N *=* *100. Formulated differently, an admissible secondary structure annotation is a well-parenthesized string of length *N* over the alphabet Σ={(,),.}. We add the constraint that two matching parenthesis (representing a base-pair) must be separated by at least *θ* = 3 dots. Structures were drawn uniformly from this set using standard Boltzmann sampling algorithms for RNA structures [Ding and Lawrence (2003), given that the uniform case corresponds to Boltzman sampling with *β* = 0]. This constitutes what we now refer to as the RANDOM dataset.

Note that, since the structures annotating the leaves of the FILTERED_RFAM dataset belong to members of the same homology family, they tend to be more similar to each other than the structures of the RANDOM dataset. They therefore provide complementary settings when it comes to comparing our various combinations of constraint and metric.

### 5.2 Experimental protocol

**Figure 3.**
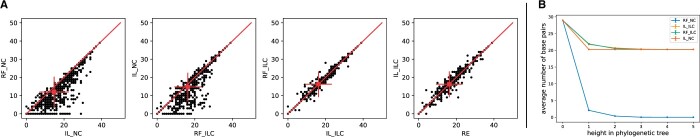
(A) Scatter plots of the number of base-pairs predicted at the root of the phylogeny by RF_NC, RF_ILC, IL_ILC, IL_NC, and RE for all families in the FILTERED_RFAM dataset. Each point is an RFAM family, with its *x*, *y*-coordinates equal to the number of predicted base-pairs at the root of the phylogeny by the respective methods. For each plot, the red square point indicates the average, its error bars being the standard deviation. As in Supplementary Fig. S6 (Supplementary Appendix), RF_NC seems to provide the least resolved results. IL_NC performs slightly better, but RF_ILC and IL_ILC yield the most base pairs. Both of them seem to fare comparably with each other, indicating that the ILC constraint (only internal leafsets from the input structures) is the deciding factor. (B) Average maximum number of base-pairs in reconstructed ancestral structures, as a function of the height of the corresponding node in the tree, over the RANDOM dataset. We observe as a general trend that the number of base-pairs in predicted ancestral structures decreases as we move up the trees. However, where RF_NC very quickly predicts empty structures at ancestral nodes, the other metric/constraint combinations (RF_ILC, IL_ILC and IL_NC) do predict nonempty structures. Remarkably, IL_NC does so without any constraint.

For both the FILTERED_RFAM and RANDOM datasets, we infer ancestral structures under the combination of metrics and constraints RF_NC, IL_NC, IL_ILC, and RF_ILC using our polynomial exact algorithm (RF_NC) and heuristics (IL_NC, IL_ILC, and RF_ILC, see “median-based heuristic” above). Our purpose is to assess the capacity of each method to yield *resolved* ancestral structures. As a proxy for resolution, we look at the (average and/or maximum) number of base-pairs of ancestral structures as we climb up the phylogenies. The rationale is that a reasonable feature to expect from ancestral reconstruction methods could be the conservation of the average number of base-pairs regardless of the height in the phylogeny. Our experimental results are displayed on Supplementary Fig. S6 (Supplementary Appendix) and Fig. 3A (for the FILTERED_RFAM dataset) and B (for the RANDOM dataset).

## 6 Results and discussion

### 6.1 Execution times

Table 1 reports average execution times per family on the FILTERED_RFAM dataset, for each of our methods. They are consistent with the hierarchy of theoretical time complexity. The IL_NC approach is unsurprisingly slower and the RF_NC faster since it does not require exploring medians until convergence. The IL_ILC and RF_ILC do have to apply this step, but are reasonably fast owing to their faster dynamic programming algorithm. The RE implementation is the slowest.

**Table 1.** Average execution times per family of each combination of metric and constraint, on the FILTERED_RFAM dataset.

Metric+constraint	Avg. time per family (seconds)
RF_NC	0.26
IL_NC	10.5
IL_ILC	1.39
RF_ILC	1.51
RE	45.9

### 6.2 Resolution of ancestors

Fig. 3A and B, and Supplementary Fig. S6 (Supplementary Appendix) aim at representing the effect of the choice of metric and constraint on the *resolution* of the Small Parsimony problem. By resolution, we mean the tendency to infer ancestors with detailed characteristics, in spite of divergences between leaf annotations. We use the number of predicted base-pairs as a proxy for the resolution of ancestral structures.

### 6.3 Low resolution for RF_NC

The Robinson-Foulds metric is a popular choice for comparing phylogenetic trees (Robinson and Foulds 1981). When it comes to RNA structures however, our results on Supplementary Fig. S6 (Supplementary Appendix), Fig. 3A and B show that solving Small Parsimony under this distance leads to reconstructed ancestral structures with few base-pairs. The results are particularly striking for the RANDOM dataset (Fig. 3B). This makes sense in the light of the fact that in the RANDOM dataset, structures at leaves tend to be more distant to each other, compared to FILTERED_RFAM dataset. This low resolution of RF_NC might be partially due to the systematic preference of 0 (absence of a descendant leaf-set) over 1 (presence) at the root of the phylogenies or, perhaps more likely, to the absence of constraints. In particular, as formulated in Proposition 4, *any* set of nonconflicting descendants leaf-sets yields a valid structure. If this set is small, the corresponding structure has few base-pairs.

### 6.4 Intermediate resolution for IL_NC

Figure 3 and Supplementary Fig. S6 (Supplementary Appendix) show that for the FILTERED_RFAM dataset, intermediate levels of resolution are achieved with IL_NC (higher than RF_NC, lower than IL_ILC and RF_ILC). This may be due to the fact that the IL distance favors the presence of *loops* from the input structures, and a loop is present only if *all the base-pairs defining it* are present.

### 6.5 Highest resolution with the ILC constraint

The highest resolution is obtained by enforcing the ILC constraint (only internal leaf-sets from the input structures). A possible explanation for this fact is that, in the case of high divergence between some of the leaf solutions, the best median solution may be an empty structure. The ILC constraint forbids this possibility, and enforces higher resolution at ancestral nodes. This occurs regardless of the choice of metric (RF or IL), signaling the strength of the ILC constraint. As for the RE method, given its restriction (Section 4.3) to leaf annotations for internal nodes, it unsurprisingly yields high resolution.

### 6.6 Comparison of SP costs

**Figure 4.**
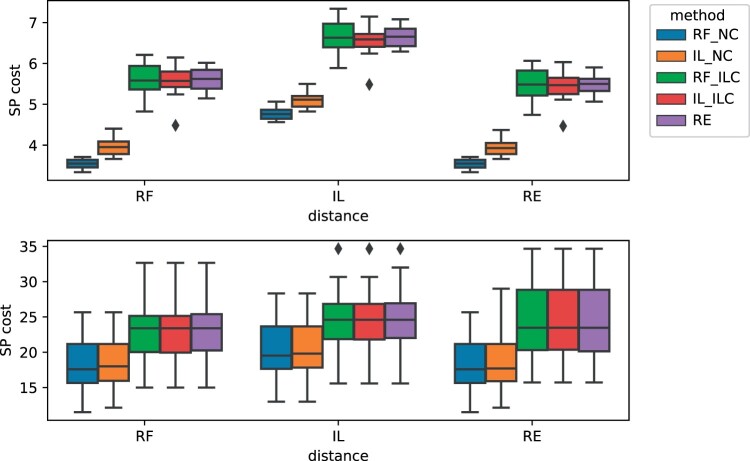
Distribution of the Small Parsimony (SP) costs obtained over the FILTERED_RFAM (bottom) and the RANDOM dataset with random structures of length 30 (top), by each method with respect to all three distances. The Small Parsimony cost is the sum of distances (either RF, IL, or RE) over each edge of a phylogeny. To allow averaging over several RFAM families, costs are divided by the number of edges in the phylogeny.

Figure 4 shows the distribution of SP costs (the sum of distances on the branches) for our datasets. On the FILTERED_RFAM dataset, we restricted this analysis to the 20 families that exhibit the most divergence at the leaves. The unconstrained predictions typically achieve a lower SP cost than their constrained counterparts, which is not surprising since they are allowed a larger solution space. There is little difference in terms of cost between the constrained versions. There appears to be an inevitable trade-off: the constrained versions offer better resolution, but at the price of a higher SP cost. On the RANDOM dataset, the differences in cost are even more pronounced. Notably, structures inferred by the unconstrained RF distances achieve lower SP cost, again illustrating its relationship with the level of resolution.

## 7 Conclusion

Investigating the evolution of RNA structures provides information about their functional adaptations and their role in the evolution of organisms. In particular, it is expected to shed light on the RNA World hypothesis.

In this work, we have established novel foundations for the reconstruction of ancestral RNA structures by borrowing several ideas from phylogenetics. Combining research in structural RNA and phylogenetics is promising since, compared to prior work, our algorithmic framework allows comparing any set of aligned RNA secondary structures, whether the alignment displays crossing base pairs, the structures are similar or distant, homologs or not. Moreover, instead of inferring constrained ancestral RNA sequences, we predict the evolutionary history of the structures. Furthermore, we introduced the novel IL distance, which compares structural motifs instead of solely base pairs. To our knowledge, this distance had not been considered previously, and further exploration is required to understand its potential. Moreover, our implementation was able to assess the resolution on RFAM and randomized data.

Our work paves the way for several future research directions. In terms of algorithms, the complexity of the Small Parsimony problems under IL_NC, IL_ILC, and RF_ILC is still open. The RE Median and Small Parsimony problems are also open, even for designing heuristics. We mention in passing that the IL median problem is also open for *unordered* trees and may be of independent interest. In practice, it remains to improve the performance of our algorithms, especially the IL algorithms that have a cubic or quintic dependency on *n*. Also, the results (Fig. 4) shows that RF_NC is more parsimonious than the IL_NC and IL_ILC distances, but perhaps with better heuristic for the latter, we could obtain similar SP costs with better resolutions. Furthermore, the problems and methods introduced here only consider the structure *P* of an RNA secondary structure (*S*, *P*). In future extensions, the RNA sequences can be involved either by considering a distance metric that incorporates both the sequence and the structure to reconstruct parsimonious ancestral sequences and structures, or by using the resulting parsimonious ancestral structures as a constraint to subsequently compute parsimonious ancestral RNA sequences under a structurally constrained sequence evolution model. This will allow us to compare the results of our methods against ancestral RNA sequence reconstruction methods such as achARNement (Tremblay-Savard *et al.* 2016). Moreover, we have studied the restricted version of the problems where RNA structures are not subject to nucleotide indel events, thus considering RNA sequences of the same length. Future algorithmic extensions of the problems will include considering a gapped alignment as input. This will allow our framework to handle multiple RNA families, for instance RNA families exhibiting structural differences but yet grouped into clans. Other problems of interest are to seek the optimal median or the optimal small parsimony assignment, as well as the alignment when the alignment is not provided as input. Finally, another possible direction for future work is the inclusion of pseudoknots into the input structures, for instance thanks to treewidth-based techniques (Jiang *et al.* 2002, Rinaudo *et al.* 2012, Marchand *et al.* 2022).

## Supplementary Material

btae229_Supplementary_Data
