# Supplementary material for: Median and small parsimony problems on RNA trees
Source: Bioinformatics. 2024 Jun 28;40(Suppl 1):i237–46. doi: 10.1093/bioinformatics/btae229 (PMC11256950; doi:10.1093/bioinformatics/btae229)
Supplement: btae229_Supplementary_Data [file btae229_supplementary_data.pdf]

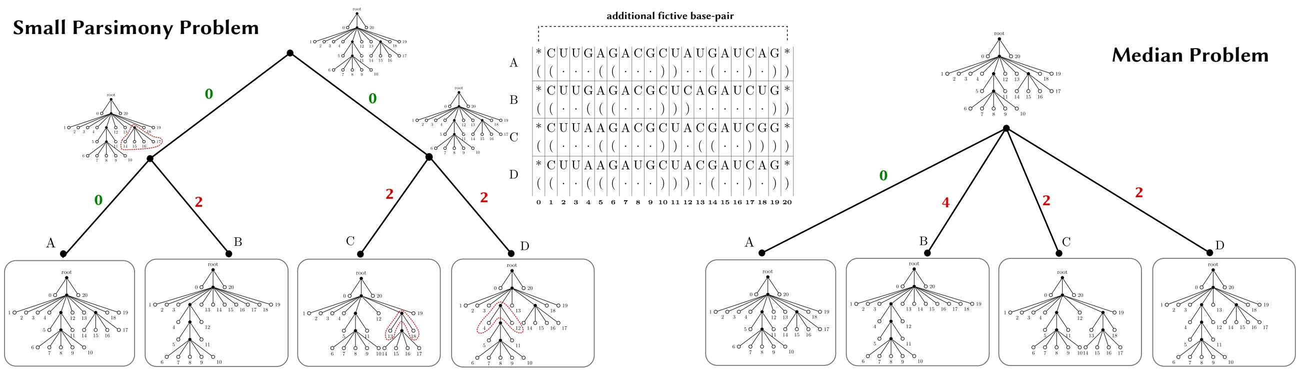

Fig. 5. Annotation of Figure 2 with possible optimal solutions for the small parsimony and median problems, under the RF_NC distance. In the case of the small parsimony problem (left), the descendant leaf-sets that are responsible for non-zero edge costs have been highlighted in red. For the median problem (right), the majority rule (Proposition 5) has been applied. Here, one of the input structures is an optimal median. This is not always the case.

Appendix

The detailed proofs of our results can be found below.

Proof of Proposition 1

Given an RNA secondary structure $R = (S, P)$ such that S has length n, $L(R) = [0, n+1]$ and for any internal node $(i, j) \in I(R)$, the leftmost and rightmost children of (i, j) are the leaf nodes i and j by construction. Therefore, $T(R)$ is an RNA tree with leaf set $[0, n+1]$.

Given an RNA tree T with leaf set $[0, n+1]$, $P = I(T) - (0, n+1)$ satisfies the condition that any two distinct pairs (i, j) and (k, l) in P are either nested, or $i < j < k < l$, or $k < l < i < j$. Therefore, if S is a string such that for any pair $(i, j) \in P$, $S_i S_j$ is either a Watson-Crick or a Wobble base pair, then $R = (S, P)$ is an RNA secondary structure.

Proof of Proposition 2

In an RNA tree, a base pair (i, j) corresponds to the DL $[i, j]$, and vice versa. Since the two concepts are equivalent, the distances based on the symmetric differences are also equivalent.

Proof of Proposition 3

The cost function heavily penalizes the mapping of different nodes of T_1 and T_2, i.e different base pairs of R_1 and R_2. Therefore, an optimal mapping cannot contain a pair of mapped nodes that are different. The optimal mapping minimizing $cost_c(M)$ is then $M = \{((i, j), (i, j)) \mid (i, j) \in I(R_1) \cap I(R_2))\}$, and thus $cost_c(M) = |I(R_1)| + |I(R_2)| - 2 \times |I(R_1) \cap I(R_2)| = |I(R_1) \Delta I(R_2)|$.

Proof of Proposition 5

We claim that $\mathrm{DL}^+(T_1, \ldots, T_p)$ must be conflict-free. Indeed, given two intervals X and Y in $\mathrm{DL}^+(T_1, \ldots, T_p)$, since they are both present in strictly more than half of the input trees, then there must be a tree T_i in which they are both displayed. By Proposition 4, X and Y cannot be conflicting. Since $L \in \mathrm{DL}^+(T_1, \ldots, T_p)$, by Proposition 4, there exists an RNA tree T^* such that $\mathrm{DL}^+(T_1, \ldots, T_p) = \mathrm{DL}(T^*)$.

Now, consider T an RNA tree displaying a DL L' which is not displayed by more than half of the input trees, i.e. $L' \notin \mathrm{DL}^+(T_1, \ldots, T_p)$. Say $L' = \mathrm{DL}(x)$ for some internal node x of T. Consider now T' obtained from T by merging the node x with its parent, effectively removing L' from the set of descendant leafsets. We then have: $\mathrm{Mcost}(T') = \mathrm{Mcost}(T) + |\{i \mid L' \in \mathrm{DL}(T_i)\}| - |\{i \mid L' \notin \mathrm{DL}(T_i)\}|$ Since L' is not displayed by more than half of the input trees, then $\mathrm{Mcost}(T') \leq \mathrm{Mcost}(T)$. Note that the equality holds if L' is displayed by exactly half of the trees. So, by merging all nodes of T whose descendant leafsets are not in $\mathrm{DL}^+(T_1, \ldots, T_p)$ with their parents, we can obtain an RNA tree T" that displays only DL that are in $\mathrm{DL}^+(T_1, \ldots, T_p)$ and such that $\mathrm{Mcost}(T") \leq \mathrm{Mcost}(T)$. In addition, if the tree T" displays only a strict subset of $\mathrm{DL}^+(T_1, \ldots, T_p)$, i.e not all DLs in $\mathrm{DL}^+(T_1, \ldots, T_p)$, then $\mathrm{Mcost}(T^*) < \mathrm{Mcost}(T")$. Therefore, T^* is an optimal solution of the RF_NC Median Problem.

To reconstruct T^*, one can first enumerate and count the set of all DLs in time $O(pn^2)$, as each of the $O(p)$ trees has $O(n)$ DLs. Each time a DL is encountered, we can update its count in a hash table, with hashing time $O(n)$ per DL (since each DL can have linear size). Hence the counting phase takes time $O(pn^2)$. Finding the majority DLs can be achieved by checking the count of the $O(pn)$ DLs, and once they have been obtained, reconstructing the tree can be done in time $O(n^2)$ using known approaches (e.g. Gusfield (1991)). The complexity is dominated by the counting phase.

Proof of Lemma 1

($\Rightarrow$) Since every leaf of T has a unique parent, each leaf belongs to a unique IL and this $IL(T)$ forms a partition of $[i, j]$. Moreover, since each internal node u of an RNA tree represents a base pair (x, y), each set in $IL(T)$ has at least two elements.

As for the conflict-free property, consider two internal nodes u, v. If u and v are incomparable (none is an ancestor of the other), then $DL(u) = [k_u, l_u], DL(v) = [k_u, l_u]$ satisfies $[k_u, l_u] \cap [k_v, l_v] = \emptyset$. Since $IL(u) \subseteq DL(u), IL(v) \subseteq DL(v)$, no conflict is possible. So suppose w.l.o.g that u is an ancestor of v. Since T is an ordered tree, all leaves that descend from v (including $IL(v)$), are contained in the same gap of $IL(u)$, from which it is easy to see that no conflict is possible.
($\Leftarrow$) Conversely, let $\mathcal{I}$ be a structural partition of $[i, j]$, with i, j in the same set of $\mathcal{I}$. An RNA tree can be obtained by creating an internal node for each element of $\mathcal{I}$, creating a leaf for each element x in $[i, j]$, and connecting x the unique internal node whose IL contains x. A node associated to IL $I \in \mathcal{I}$ is made the parent of a node associated to IL $J \in \mathcal{I}$ if $I \preccurlyeq J$ and there is no I' such that $I \preccurlyeq I' \preccurlyeq J$. It is not hard to see that this creates a tree rooted at the IL that contains i, j, and that the

conflict-free property allows ordering the children of nodes as required by RNA trees.

Proof of Lemma 2

By definition, M must minimize $\sum_{1\leq i\leq p} |\text{IL}(T_i)\Delta\text{IL}(M)|$. It can be rewritten as such:

$$\begin{aligned}\sum_{i=1}^{p} |\text{IL}(T_i)\Delta\text{IL}(M)| &= \sum_{i=1}^{p}(|\text{IL}(T_i)\setminus\text{IL}(M)| + |\text{IL}(M)\setminus\text{IL}(T_i)|)\\ &= \sum_{i=1}^{p}(|\text{IL}(T_i)| - |\text{IL}(T_i)\cap\text{IL}(M)|) + \\ &\sum_{I\in\text{IL}(M)} |\{i|I\notin IL(T_i)\}|\\ &= \sum_{i=1}^{p}|\text{IL}(T_i)| - \sum_{I\in\text{IL}(M)}|\{i \mid i\in\text{IL}(T_i)\}| + \\ &\sum_{I\in\text{IL}(M)} |\{i \mid I\notin\text{IL}(T_i)\}|\end{aligned}$$

Given that $\sum_{i=1}^{p} |\{\text{IL}(T_i)\}|$ does not depend on the choice of M, finding a median is equivalent to minimizing the expression as stated.

Proof of Theorem 1

For convenience, we recall that $c[i,j]$ is the minimum cost of a structural partition of $[i,j]$ under the ILC constraint, and $\hat{c}[i,j]$ the same cost but without the constraint. In the main text, we provided the recurrences

$$c[i,j] = \min_{\substack{I\in\mathcal{I}_{i,j}\\ \text{s.t } i\in I}}\left[cost_{IL}(I) + \sum_{[x,y]\in\Gamma(I)\cup\{[\max(I)+1,j]\}} c[x,y]\right]$$

and

$$\hat{c}_1[i,j] = \min_{\substack{I\in\mathcal{I}_{i,j}\\ \text{s.t } i\in I}}\left[cost_{IL}(I) + \sum_{[x,y]\in\Gamma(I)\cup[\max(I)+1,j]} \hat{c}[x,y]\right]$$

$$\hat{c}_2[i,j] = \min_{i<k\leq j}(p + \hat{c}[i+1,k-1] - \alpha(G_{k+1,j}))$$

$$\hat{c}[i,j] = \min(\hat{c}_1[i,j],\hat{c}_2[i,j])$$

We prove here the correctness of the recursions, and conclude with a complexity analysis. Let i,j be integers. We show that our recurrences for $c[i,j]$ and $\hat{c}[i,j]$ correctly compute the minimum cost of a structural partition (with the ILC constraint in the former case).

As a base case for the c entries, $\mathcal{I}_{i,j} = \emptyset$, no structural partition under the ILC constraint is possible and putting $c[i,j]=\infty$ is correct. For the $\hat{c}$ entries, if $i=j$ no structural partition is possible because so set of a partition of $\{i\}$ can have two elements, and so $\hat{c}[i,j]=\infty$ is correct. For $i<j$, $\hat{c}[i,j]=0$ can be seen as correct since the empty partition has cost 0.

Now assume inductively that $c[i',j']$ and $\hat{c}[i',j']$ are correct for any $[i',j']\subset[i,j]$. We argue that c and $\hat{c}$ are at most the cost of an optimal structural partition, then at least its cost.

($\leq$) Let S be a structural partition of $[i,j]$ of minimum cost (we handle both cases of S satisfying ILC or not in the same proof). Since S is a partition, there must be some $I\in S$ containing i. Note that since S is conflict-free, any IL other than I in S must be in a gap of I, or must be contained in $[\max(I)+1,j]$. Moreover, the sets of S contained in such a $[x,y]\in\Gamma(I)\cup\{[\max(I)+1,j]\}$ must form a partition of $[x,y]$.

If S is required to satisfy the ILC constraint, then the cost of S is at least $cost_{IL}(I)$, plus the sum of $c[x,y]$ for every such $[x,y]$ (by induction). Since our recurrence for $c[i,j]$ minimizes over every $I\in\mathcal{I}_{i,j}$, it will, in particular, consider I at some point, and thus $c[i,j]$ is no more than the cost of S.

So suppose that S is not required to satisfy the ILC constraint. If $I\in\mathcal{I}_{i,j}$, by the same arguments as above, $\hat{c}_1[i,j]$ as computed above is no more than the cost of S, and thus the same holds for $\hat{c}[i,j]\leq\hat{c}_1[i,j]$. On the contrary, if I is not an input leafset, then $cost_{IL}(I)=p$. Let k be the minimum integer in $I\setminus\{i\}$ (which exists since sets of S have size at least two). Then $[i+1,k-1]$ is a gap of I and the subsets of S in $[i+1,k-1]$ form a structural partition of cost at most $\hat{c}[i+1,k-1]$, by induction. Moreover, let $[x_1,y_1],\ldots,[x_k,y_k]$ be the other gaps of I, including $[\max(I)+1,j]$ if non-empty. Then S restricted to any of these intervals forms a structural partition, and the sum of costs of these is at most $\sum_{i=1}^{k}\hat{c}[x_i,y_i]$. These intervals form an independent set of $G_{k+1,j}$ with cost minus 1 times this sum, and thus $-\alpha(G_{k+1,j})$ is no more than this sum. Since the expression of $\hat{c}_2[i,j]$ minimizes over every possible k, $\hat{c}_2[i,j]$ is no more than the cost of S, and thus $\hat{c}[i,j]$ as well.

($\geq$) For the other direction, first consider the entry $c[i,j]$ when the ILC constraint is present. Let I be any subset of $\mathcal{I}_{i,j}$ containing i. Suppose that the expression in large brackets for $c[i,j]$ is ∞. Then there is some gap $[x,y]\in\Gamma(I)\cup\{[\max(I)+1,j]\}$ such that $c[x,y]=\infty$. Since this value is correct by induction, this means that there is no structural partition of $[x,y]$, and thus none for $[i,j]$ either when I is in the partition (because it is impossible to partition one of its gaps). This means that if *every* $I\in\mathcal{I}_{i,j}$ yields ∞, there cannot be a structural partition of $[i,j]$ with the ILC condition, and we will correctly put $c[i,j]=\infty$.

Assume otherwise, and suppose that I minimizes the expression of $c[i,j]$. For each gap $[x,y]$ of I, including $[\max(I)+1,j]$ if non-empty, let $S_{x,y}$ be an optimal structural partition of $[x,y]$. By induction, its cost is $c[x,y]$. Taken together, I and the union of the $S_{x,y}$ are easily seen to be a structural partition of cost $cost_{IL}(I)$, plus the sum of $c[x,y]$ for each gap $[x,y]$. It thus forms a valid solution for the sub-problem associated, and may not be better than optimal, therefore our recurrence for $c[i,j]$ is at most the cost of an optimal solution for $[i,j]$.

Next, suppose that the ILC constraint is not present. Note that $\hat{c}[i,j]<\infty$. This is because for $i<j$, the entry for $\hat{c}_2[i,j]$ can choose $k=i+1$ and choose an empty independent set, which corresponds to a solution of cost p. Since this is a possibility for $\hat{c}[i,j]$, the latter is p or better. If $\hat{c}[i,j]=\hat{c}_1[i,j]$, then one can obtain a valid structural partition of $[i,j]$ by taking the I that minimizes the expression, and the optimal solution for each gap. As in the previous case, this gives a solution of cost at most $\hat{c}_1[i,j]$. Since it cannot be better than optimal, we get that $\hat{c}[i,j]=\hat{c}_1[i,j]$ is at most the cost of an optimal structural partition.

So suppose that $\hat{c}[i,j]=\hat{c}_2[i,j]$. Let k be the index that minimizes its expression, and let R be a maximum independent set of $G_{k+1,j}$. Let $I=[i,j]\setminus(\{[i+1,k-1]\}\cup\bigcup_{[x,y]\in R}[x,y])$. Note that I has size at least two since it contains i and k. The partition obtained from the union of I (of cost p), of an optimal structural partition of $[i+1,k-1]$ (of cost $\hat{c}[i+1,k-1]$), and of an optimal structural partition of each $[x,y]\in R$ (of total cost $-\alpha(G_{k+1,j})$), yields a structural partition of $[i,j]$ of cost $\hat{c}_2[i,j]$. Since it cannot be better than optimal, we get that

$\hat{c}[i,j] = \hat{c}_2[i,j]$ is at most the cost of an optimal structural partition.

Having completed both sides of the inequality, we see that $c[i,j]$ (resp. $\hat{c}[i,j]$) correctly computes the minimum cost of a constrained (resp. unconstrained) structural partition of $[i,j]$.

To finish the argument, we need to argue that $c[0,n+1]$ and $\hat{c}[0,n+1]$ correspond to the cost of an optimal median tree. If $0, n+1$ are in the same set of the structural partitions correspond to those c and $\hat{c}$ entries, then this holds by Lemma 1. It is not hard to see that some optimal structural partition must put $0, n+1$ in the same set, since the IL $\{0, n+1\}$ is in every input tree. We omit the details.

As for the complexity, consider the time required to compute every entry $c[i,j]$. There are $O(n^2)$ entries to fill. Each of them requires iterating over every $I \in \mathcal{I}_{i,j}$ that contains i. Note that each input tree has exactly one IL containing i, so there are p entries to enumerate. For each I in this enumeration, we can compute $cost_{IL}(I)$ and the sum in time $O(n)$ (assuming constant time access to the $c[x,y]$ entries). The overall complexity is therefore $O\left(n^2 \cdot pn\right) = O(pn^3)$.

Next, consider the time to calculate the $O(n^2)$ entries $\hat{c}[i,j]$. As before, $\hat{c}_1[i,j]$ takes time $O(pn)$. Then, $\hat{c}_2$ iterates over $O(n)$ values of k. Each of them makes a call to a solver for the independent set problem over $O(n^2)$ intervals. It is known that such an independent set can be found in time $O(n^2)$ if the input intervals are given in right-end sorted order, which is easy to construct here (Hsiao *et al.*, 1992). Thus, the time spent for one $\hat{c}_2$ entry is $O(n^3)$. One $\hat{c}$ entry takes time $O(pn + n^3)$, for a total time of $O(pn^3 + n^5)$.

Pseudo-code of IL_ILC (and RF_ILC) median: Algorithm 1

Algorithm 1 Folding-like, dynamic programming algorithm for computing the IL_ILC (and RF_ILC) median of a set of input trees.Pseudo-code of

Input: structural trees $T_1, \ldots, T_p$ over $[0, n+1]$, a boolean input_only

Output: An RNA tree M minimizing $\sum_{i=1}^{p} d(T_i, M)$. If input_only is true, then M may only contain leaf-sets from the input trees

function IL_MEDIAN($T_1, \ldots, T_p$, input_only):
    $\hat{c} = \{\}$ ▷ // initializing DP table
    optimum = optimal_score(0, n+1) ▷ filling DP table
    M = tree_from_leafsets(backtrace(0, n+1))
    return M
end function

function optimal_score(i, j)
    if (i,j) in c **then return** $c[i,j]$
    end if
    $\hat{c}[i,j] = +\infty$

    // *Computing* $\hat{c}_1[i,j]$
    for I in $\cup_{i=1}^{p} \mathcal{IL}(T_i)$ if I contains i **do**
        score_with_I = $|\{i \mid I \notin \mathcal{IL}(T_i)\}| - |\{i \mid I \in \mathcal{IL}(T_i)\}|$
        $+\sum_{h \in \texttt{holes}(I)}$ optimal_score(i_h, j_h)
        $\hat{c}[i,j] = \min\left(\hat{c}[i,j], \text{score\_with\_I}\right)$
    end for

    if not ILC **then**
        // *Computing* $\hat{c}_2[i,j]$
        for $k \in [i+1, j]$ **do**
            α = mwIS_interval_graph($G_{k+1,j}$)
            // *line above requires* $\hat{c}[u,v]$ *for all* $i < u < v < j$
            $\hat{c}[i,j] = \min\left(\hat{c}[i,j], p + \hat{c}[i+1, k-1] - \alpha\right)$
        end for
    end if
    return $\hat{c}[i,j]$
end function

function backtrace(i, j)
    for I in $\cup_{i=1}^{p} \mathcal{IL}(T_i)$ if I contains i **do**
        score_with_I = $|\{i \mid I \notin \mathcal{IL}(T_i)\}|$
        $+\sum_{h \in \texttt{holes}(I)} c[i_h, j_h]$
        if score_with_I = $c[i,j]$ **then**
            return $[I]$ + concatenate({backtrace(x, y) | $(x,y) \in$ holes(I)})
        end if
    end for
    if not ILC **then**
        for $k \in [i+1, j]$ **do**
            α = mwIS_interval_graph($G_{k+1,j}$)
            if $\hat{c}[i,j] = p + \hat{c}[i+1, k-1] - \alpha$ **then**
                // mwIS: *a maximum weight IS of* $G_{k+1,j}$
                return $[\{i,k\} \cup [k+1, j] \setminus$ mwIS$]$
                +backtrace($i+1, k-1$)
                +concatenate ({backtrace(x, y) | $x, y \in$ mwIS})
            end if
        end for
    end if
end function

Proof of Theorem 2

Recall that our input is a set of trees $T_1, \ldots, T_p$, and phylogeny $\mathbb{T}$ with p leaves, where the i-th leaf is assigned to the RNA tree T_i. In what follows, we will denote $\mathcal{DL} = \bigcup_{i=1}^{p} DL(T_i)$ as the set of DLs that occur in at least one tree. For any such $c \in \mathcal{DL}$ and node $u \in V(\mathbb{T})$ of the input phylogeny, we define the bottom-up sets $B(c,u)$ in the following way. If u is a leaf assigned to tree T, then $B(c,u) = \{1\}$ if $c \in DL(T)$, and $B(c,u) = \{0\}$ otherwise. If u is not a leaf, then let $children(u)$ be the set of children of u in $\mathbb{T}$. We denote by $N_0(c,u)$ (resp. $N_1(c,u)$) the number of children v of u satisfying $B(c,v) = \{0\}$ (resp. $B(c,v) = \{1\}$). Then, if $N_0(c,u) > N_1(c,u)$, we assign $B(c,u) = \{0\}$ if $N_1(c,u) > N_0(c,u)$, $B(c,u) = \{1\}$, and otherwise, $B(c,u) = \{0,1\}$.

Note that $B(c,u)$ is as computed in the Fitch-Hartigan approach for binary characters. As stated in (Semple and Steel, 2003, Theorem 5.2.1), $B(c,u)$ contains 1 (resp 0) if some optimal solution of the subtree rooted at u puts c (resp. does not put c) in the RNA tree inferred at u. Once the bottom-up sets have been built, final assignments $F(c,u)$, with $c \in \mathcal{DL}$ and $u \in V(\mathbb{T})$ a node of the input tree, are computed in the following way. If u is the root, then if $0 \in B(c,u)$ we set $F(c,u) = 0$, and $F(c,u) = 1$ otherwise. If u is not the root, then let w be its parent. If $B(c,u) = \{0,1\}$ then $F(c,u) = F(c,w)$. Otherwise, we have $B(c,u) = \{x\}$ for $x = 0$ or 1, and we put $F(c,u) = x$. Again, this is the same as in the Fitch-Hartigan method, except that we give a priority to 0 if there is a choice at the root (in which case the choice propagates to the descendants as long as they have 0 in their possible assignment). We adapt here the Lemmas of Feijao and Meidanis (2011). The main difference is that we deal with DLs instead of adjacencies, and we also deal with a possibly non-binary tree $\mathbb{T}$, but these differences are marginal. The first step is to argue that candidate states enforced by B cannot be conflicting, and the second step that the final assignments $F(c,u)$ are also free of conflicts.

Lemma 3. *Let $c, d \in \mathcal{DL}$ be two DLs in conflict and let u be a node of $\mathbb{T}$. If $B(c,u) = \{1\}$ then $B(d,u) = \{0\}$.*

Proof We prove the lemma by induction on the height of the tree. If u is a leaf, then the property is true because an RNA tree (i.e. a conflict-free set of DLs) was assigned to u as input.

Suppose that u is an internal node, and that the property is true for every child of u. Assume that $B(c,u) = \{1\}$. Then $N_1(c,u) > N_0(c,u)$, that is with respect to c, u has strictly more children with label $\{1\}$ than label $\{0\}$.

Let v be a child of u with $B(c,v) = \{1\}$. By induction, we know that $B(d,v) = \{0\}$. That is, each $\{1\}$ child of u with respect to c has a $\{0\}$ with respect to d, which lets us deduce that $N_0(d,u) \geq N_1(c,u)$. Likewise, let v be a child of u with $B(d,v) = \{1\}$. By induction, we have $B(c,v) = \{0\}$. Therefore, $N_0(c,u) \geq N_1(d,u)$. Putting the gathered inequalities together, we get

$$N_1(d,u) \leq N_0(c,u) < N_1(c,u) \leq N_0(d,u)$$

and, since $N_1(d,u) < N_0(d,u)$, we put $B(d,u) = \{0\}$ as desired. □

Lemma 4. *Let $c, d \in \mathcal{DL}$ be conflicting DLs and let u be a node of $\mathbb{T}$. If $F(c,u) = 1$ then $F(d,u) = 0$.*

Proof We proceed by induction on the depth of u. As a base case, if u is the root, then $F(c,u) = 1$ only in the case where $B(c,u) = \{1\}$, which implies by Lemma 3 that $B(d,u) = \{0\}$ and therefore $F(d,u) = 0$.

Suppose that u is not the root but that the property holds for its parent w. Assume that $F(c,u) = 1$. By the definition of F, this can only occur if one of the following occurs:

- $B(c,u) = \{1\}$, in which case $B(d,u) = \{0\}$ by Lemma 3, and $F(d,u) = 0$ by definition;
- $B(c,u) = \{0,1\}$ and $F(c,w) = 1$. In this case we know $B(d,u) \neq \{1\}$, as otherwise Lemma 3 would imply $B(c,u) = \{0\}$. Therefore, $0 \in B(d,u)$. By our induction hypothesis, $F(c,w) = 1$ implies $F(d,w) = 0$, which in turn implies $F(d,u) = 0$.

In all cases, $F(c,u) = 1$ does imply $F(d,u) = 0$. □

Once B and F are computed, we can simply assign to each $u \in V(\mathbb{T})$ the RNA tree whose set of DLs are those $c \in \mathcal{DL}$ for which $F(c,u) = 1$. This is possible since, by Proposition 4, any set of conflict-free DLs can be turned into an RNA tree. Let us first mention that the F assignments produce an optimal solution. Indeed, because the set of assigned DLs are those obtained from the Fitch-Hartigan approach, we know that F yields a solution that minimizes the $0-1$ or $1-0$ changes on the branches, which is equivalent to minimizing the symmetric difference. We refer to (Semple and Steel, 2003) and (Feijao and Meidanis, 2011) for more details on the optimality of the approach. Since the characters $c \in \mathcal{DL}$ are restricted to the descendant leaf-sets of the input structures, the output is DLC by construction. We argue that it is also a solution to the RF_NC case. Indeed, if in a solution a descendant leaf-set L that is not found at the leaves of $\mathbb{T}$ is found in a subset S_L of the vertices of $\mathbb{T}$, then a gain of cost function of $|E(S_L, V(\mathbb{T}) \backslash S_L)|$ (the number of edges with an end-point in S_L and one outside of S_L) is obtained by removing L everywhere it appears. The present Theorem therefore handles both the RF_NC and RF_DLC cases.

In terms of complexity, for each $c \in \mathcal{DL}$, computing one $B(c,u)$ entry requires iterating over the children of u. Thus, the time needed to compute all $B(c,u)$ entries is proportional to the sum of number of children of nodes u in $\mathbb{T}$, which is $O(|V(\mathbb{T})|)$. The time to compute all the F entries is no more than for B. Thus, the total time for the B and F phase is $O(|C||V(\mathbb{T})|)$, which is $O(pn|V(\mathbb{T})|)$ (with p the number of RNA trees and n the number of leaves). For each $u \in V(\mathbb{T})$, we can reconstruct the RNA tree to assign to it in time $O(pn)$ by listing the DLs c with $F(c,u) = 1$, and building the tree in time $O(n)$ (this is easy to do if we assume that DLs are represented as intervals $[i,j]$, we omit the details). Thus, the reconstruction phase does not take more time than the B and F phase. The total reconstruction time is therefore $O(pnV(\mathbb{T}))$.

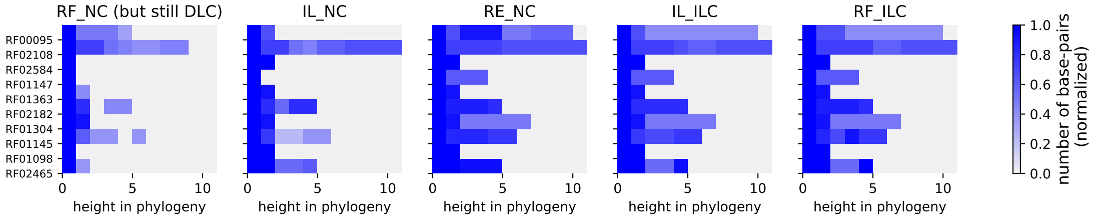

Fig. 6. Maximum number of base-pairs as a function of the height of nodes in the phylogeny, for a selected set of 10 maximally-divergent RFAM families. The number of base-pairs are normalized, for each family, by the maximum number of base-pairs over the structures annotating the leaves. The selected "maximally-divergent" families are the ones maximizing the sum of distances over pairs of leaves, as measured by the Internal-Leafset distance (Definition 4). Solving Small Parsimony under the metric/constraint combination RF_NC tends to yield ancestral structures with few base-pairs, as we move up the phylogenies. While also unconstrained, IL_NC tends to predict more base-pairs than RF_NC in ancestral structures. Being constrained to use only internal-leafsets from the input structures, IL_ILC and RF_ILC predict the most resolved ancestral structures, as per the criteria of the number of base-pairs. The score function difference (IL vs. RF) does not seem to have more than marginal impact. Note that RF_NC is DLC (only descendant leaf-sets from the input structure) so imposing this constraint would not help get more resolution.

References

Gusfield, D. (1991). Efficient algorithms for inferring evolutionary trees. *Networks*, **21**(1), 19–28.
